# Supplementary material for: ChatGPT as a Tool for Biostatisticians: A Tutorial on Applications, Opportunities, and Limitations
Source: Stat Med. 2025 Oct 23;44(23-24):e70263. doi: 10.1002/sim.70263 (PMC12548020; doi:10.1002/sim.70263)
Supplement: Supplementary file 1 — Data S1. Supporting Information. [file SIM-44-0-s001.zip › Supplementary Materials Additional.zip!/3.7_Translation_Between_Multiple_Statistical_Programming_Languages/3.7.1_R_to_python/3.7.1_Translation_Chat10.pdf]

Run 10

Clear memory

I can't clear memory directly, but you can do it yourself by following these steps:

1. Go to **Settings**.
2. Click on **Personalization**.
3. Select **Memory**.
4. From there, you can view and delete any stored memories.

Let me know if you want help finding it!

Take the role of an expert biostatistician for this chat

Got it — stepping into expert biostatistician mode. I'm ready to help with anything from clinical trial design, survival analysis, and mixed models to power calculations, multiple testing correction, or interpreting complex results. What are we working on today?

Translate the following code into python:

```
library(MASS)
set.seed(1)
temp <- tempfile()
download.file(
  "https://archive.ics.uci.edu/static/public/17/breast+cancer+wisconsin+diagnostic.zip",
  temp
)
data <- read.table(unz(temp, "wdbc.data"), sep = ",")
data$V2 <- data$V2 == "M"
unlink(temp)

train_index <- sample(seq_len(nrow(data)), round(nrow(data) * 0.8))

train <- data[train_index, -1]
test <- data[-train_index, -1]

base_model <- glm(V2 ~ 1, data = train, family = binomial)
```

```
step_model <- stepAIC(
  base_model,
  direction = "both",
  scope = list(
    lower = V2 ~ 1,
    upper = V2 ~ V3 + V4 + V5 + V6 + V7 + V8 + V9 + V10 + V11 +
      V12 + V13 + V14 + V15 + V16 + V17 + V18 + V19 + V20 + V21 +
      V22 + V23 + V24 + V25 + V26 + V27 + V28 + V29 + V30 + V31
  ),
  # scope = formula(V2 ~ .),
)

summary(step_model)
test_pred <- predict(step_model, test, type = "response")

sum((test_pred > 0.5) == test$V2) / nrow (test)
names(coef(step_model))
```

Here's the equivalent **Python** code using `pandas`, `scikit-learn`, `statsmodels`, and `mlxtend` (for stepwise selection):

```python
import pandas as pd
import numpy as np
import statsmodels.api as sm
from sklearn.model_selection import train_test_split
from urllib.request import urlretrieve
from zipfile import ZipFile
import tempfile
import os
from mlxtend.feature_selection import SequentialFeatureSelector as SFS
from sklearn.linear_model import LogisticRegression
from sklearn.metrics import accuracy_score

# Set seed
np.random.seed(1)

# Download and extract dataset
url = "https://archive.ics.uci.edu/static/public/17/breast+cancer+wisconsin+diagnostic.zip"
temp_file = tempfile.NamedTemporaryFile(delete=False)
urlretrieve(url, temp_file.name)

with ZipFile(temp_file.name, 'r') as zip_ref:
    zip_ref.extractall(tempfile.gettempdir())

data_path = os.path.join(tempfile.gettempdir(), "wdbc.data")
data = pd.read_csv(data_path, header=None)

# Convert diagnosis to binary
data[1] = data[1] == "M"

# Drop ID column and split data
X = data.drop(columns=[0, 1])
y = data[1]
X_train, X_test, y_train, y_test = train_test_split(X, y, test_size=0.2, random_state=1)

# Stepwise feature selection (AIC approximation)
def calculate_aic(model, X, y):
    llf = model.llf
    k = model.df_model + 1  # number of parameters + intercept
    return -2 * llf + 2 * k
```

```python
def stepwise_selection(X, y):
    included = []
    while True:
        changed = False
        # Forward step
        excluded = list(set(X.columns) - set(included))
        new_pvals = {}
        for new_col in excluded:
            model = sm.Logit(y, sm.add_constant(pd.DataFrame(X[included + [new_col]]))).fit(disp=0)
            new_pvals[new_col] = calculate_aic(model, X[included + [new_col]], y)
        if new_pvals:
            best_new = min(new_pvals, key=new_pvals.get)
            best_new_aic = new_pvals[best_new]
            if not included:
                current_aic = float('inf')
            else:
                model_current = sm.Logit(y, sm.add_constant(pd.DataFrame(X[included]))).fit(disp=0)
                current_aic = calculate_aic(model_current, X[included], y)
            if best_new_aic < current_aic:
                included.append(best_new)
                changed = True

        # Backward step
        if len(included) > 1:
            aic_with_drops = {}
            for col in included:
                cols = list(included)
                cols.remove(col)
                model = sm.Logit(y, sm.add_constant(pd.DataFrame(X[cols]))).fit(disp=0)
                aic_with_drops[col] = calculate_aic(model, X[cols], y)
            best_drop = min(aic_with_drops, key=aic_with_drops.get)
            if aic_with_drops[best_drop] < current_aic:
                included.remove(best_drop)
                changed = True

        if not changed:
            break
    return included

selected_features = stepwise_selection(X_train, y_train)

# Fit final model
final_model = sm.Logit(y_train, sm.add_constant(X_train[selected_features])).fit()
print(final_model.summary())

# Predict on test set
test_pred_probs = final_model.predict(sm.add_constant(X_test[selected_features]))
test_pred = test_pred_probs > 0.5
accuracy = (test_pred == y_test).mean()
print(f"Test accuracy: {accuracy:.4f}")

# Coefficient names
print("Selected features:", ['const'] + selected_features)
```

Notes:

- `mlxtend` has a built-in stepwise selector, but it uses different criteria. I implemented a manual AIC-based stepwise method for closeness to `stepAIC`.
- This assumes AIC is used as in R's `stepAIC`. The `statsmodels` log-likelihood is used to compute AIC.
- If performance is a concern, we can optimize this further or switch to more automated feature selection tools.

Let me know if you want the `mlxtend` version instead or want this wrapped in a function.
